# Inhibition of TGF-β/Smad3 Signaling Disrupts Cardiomyocyte Cell Cycle Progression and Epithelial–Mesenchymal Transition-Like Response During Ventricle Regeneration

**DOI:** 10.3389/fcell.2021.632372

**Published:** 2021-03-16

**Authors:** Yuanyuan Peng, Wenyuan Wang, Yunzheng Fang, Haichen Hu, Nannan Chang, Meijun Pang, Ye-Fan Hu, Xueyu Li, Han Long, Jing-Wei Xiong, Ruilin Zhang

**Affiliations:** ^1^School of Life Sciences, Fudan University, Shanghai, China; ^2^Shanghai Medical College, Fudan University, Shanghai, China; ^3^Institute of Molecular Medicine, Beijing Key Laboratory of Cardiometabolic Molecular Medicine, Peking University, Beijing, China; ^4^Department of Medicine, Li Ka Shing Faculty of Medicine, The University of Hong Kong, Pokfulam, China; ^5^School of Biomedical Sciences, Li Ka Shing Faculty of Medicine, The University of Hong Kong, Pokfulam, China; ^6^School of Basic Medical Sciences, Wuhan University, Wuhan, China

**Keywords:** ventricle regeneration, TGF-β, Smad3, cell cycle, EMT-like response

## Abstract

Unlike mammals, zebrafish can regenerate injured hearts even in the adult stage. Cardiac regeneration requires the coordination of cardiomyocyte (CM) proliferation and migration. The TGF-β/Smad3 signaling pathway has been implicated in cardiac regeneration, but the molecular mechanisms by which this pathway regulates CM proliferation and migration have not been fully illustrated. Here, we investigated the function of TGF-β/Smad3 signaling in a zebrafish model of ventricular ablation. Multiple components of this pathway were upregulated/activated after injury. Utilizing a specific inhibitor of Smad3, we detected an increased ratio of unrecovered hearts. Transcriptomic analysis suggested that the TGF-β/Smad3 signaling pathway could affect CM proliferation and migration. Further analysis demonstrated that the CM cell cycle was disrupted and the epithelial–mesenchymal transition (EMT)-like response was impaired, which limited cardiac regeneration. Altogether, our study reveals an important function of TGF-β/Smad3 signaling in CM cell cycle progression and EMT process during zebrafish ventricle regeneration.

## Introduction

Myocardial infarction (MI) is a major cause of mortality worldwide (Virani et al., 2020). Adult mammalian hearts cannot replenish the large number of cardiomyocytes (CMs) that are lost due to MI, and the loss of CMs eventually leads to heart failure (Porrello et al., 2011). In contrast, zebrafish can effectively regenerate injured hearts through the dedifferentiation, proliferation, and migration of pre-existing CMs (Poss et al., 2002; Jopling et al., 2010; Itou et al., 2012).

Cardiomyocyte proliferation after injury is usually accompanied by the disassembly of sarcomere (Zhang et al., 2013; Uribe et al., 2018) and the re-expression of cardiogenic genes, such as *nkx2.5*, *gata4*, and *hand2*, which suggests the occurrence of CM dedifferentiation (Zhang et al., 2013). Mammalian CMs retain the ability to proliferate only for a short period after birth (Naqvi et al., 2014; Mahmoud and Porrello, 2018; Ye et al., 2018; Zhu et al., 2018), and the exact mechanism by which this ability is lost is still unclear (Soonpaa et al., 1996; Patterson et al., 2017). Cell migration is also necessary for cardiac regeneration (Itou et al., 2012), and ventricular and atrial CMs can migrate into and replenish injured areas (Kikuchi et al., 2010; Zhang et al., 2013). Epicardial cells can also enter the myocardial layer through the process of epithelial–mesenchymal transition (EMT), during which the expression of EMT marker genes, such as *snail*, *twist*, and *vimentin*, are upregulated (Lepilina et al., 2006; Kim et al., 2010). However, the regulation of cell migration and how it coordinates with other cellular processes, such as cell proliferation, during cardiac regeneration remain largely unknown.

The TGF-β signaling pathway regulates the differentiation, proliferation, and migration of a variety of cell types during cardiovascular development (Azhar et al., 2003) and is also vital for cardiac regeneration (Chablais and Jazwinska, 2012; Choi et al., 2013). The expression of *tgfb1*, *tgfb2*, and *tgfb3* is upregulated in injured hearts (Chablais and Jazwinska, 2012). Activated by integrins, proteases, or matrix proteins (Chablais and Jazwinska, 2012), latent Tgfβ proteins in the extracellular matrix are released from complexes, bind to the Tgfbr2 and Tgfbr1 receptors on cell membranes, and then activate downstream Smad-dependent or Smad-independent signaling pathways (Engel et al., 1999; Yu et al., 2002; Bujak et al., 2007). The TGF-β/Smad-dependent signaling pathway is activated in a model of MI (Bujak et al., 2007), and Smad3 and Smad2 exert opposite effects in multiple cellular processes (Dogra et al., 2017; Sato et al., 2019). The mechanism by which the TGF-β/Smad3 signaling specifically affects CM proliferation and migration warrants detailed investigation.

In this study, we used a zebrafish model of ventricular ablation to determine the function of the TGF-β/Smad3 signaling pathway in cardiac regeneration. Multiple components of this pathway were upregulated/activated after injury. Treatment with SIS3, a specific inhibitor of Smad3, increased the ratio of unrecovered hearts. RNA-seq data suggested that the TGF-β/Smad3 signaling pathway was involved in multiple processes during regeneration. Further analysis revealed that the cell cycle progression and EMT-like response of CMs were impaired upon inhibition of Smad3, which led to the failure of cardiac regeneration. In conclusion, our study provides novel insights into the role of the TGF-β/Smad3 signaling pathway in regulating cardiac regeneration.

## Results

### TGF-β/Smad3 Signaling Is Activated During Zebrafish Ventricular Regeneration

**FIGURE 1:**
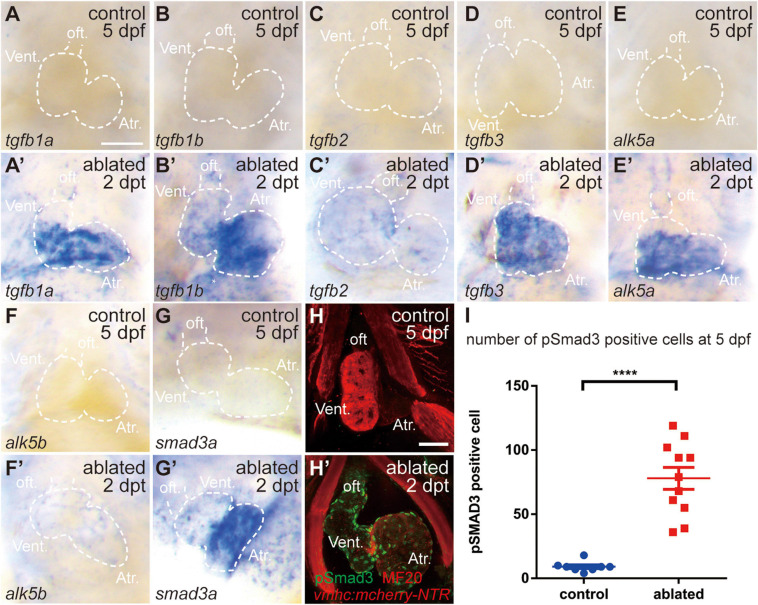
TGF-β/Smad3 signaling is activated during zebrafish ventricular regeneration. **(A–G′)** Whole-mount *in situ* hybridization showing that the expression of components of the TGF-β signaling pathway, *tgfb1a*, *tgfb1b*, *tgfb2*, *tgfb3*, *alk5a*, *alk5b*, and *smad3a*, was upregulated in the ablated hearts **(A′–G′)** compared with that in the control hearts **(A–G)** at 5 dpf/2 dpt. Dashed lines outline the hearts. **(H–H′)** Representative immunostaining images of *Tg(vmhc:mCherry-NTR)* hearts showing that phospho-Smad3 signal was increased in the ablated hearts **(H′)** than in the control hearts **(H)** at 5 dpf/2 dpt. Green, anti-pSmad3; red, MF20 (anti-MHC). **(I)** Quantification of phospho-Smad3-positive cells in the control and ablated hearts at 5 dpf/2 dpt (*N* = 8 and 11, respectively). Mean ± s.e.m., Student’s *t*-test, two-tailed, ****P < 0.0001. Scale bars, 50 μm. dpf, days post-fertilization; dpt, days post-treatment; atr., atrium; oft., out flow tract; vent., ventricle.

We first examined the involvement of the TGF-β/Smad3 signaling pathway in a model of larval ventricular regeneration using the transgenic line *Tg(vmhc:mCherry-NTR)*, which exhibits injury and death of ventricular CMs after ablation with metronidazole (MTZ) treatment at 3 days post-fertilization (dpf) (Zhang et al., 2013). Whole-mount *in situ* hybridization (WISH) showed that multiple TGF-β ligands, including *tgfb1a*, *tgfb1b*, *tgfb2*, and *tgfb3*, were strongly expressed in the ablated hearts at 5 dpf/2 days post-treatment (dpt), whereas their expressions in the control hearts were very weak (Figures 1A–D′), whose signals could not be seen under the same staining condition as ablated group but showed up after extended staining. Similarly, the gene expressions of TGF-β receptors *alk5a*, *alk5b*, and cofactor *smad3a* were also dramatically upregulated in the ablated hearts (Figures 1E–G′). Immunofluorescence staining showed that the number of cells expressing phospho-Smad3, the active form of R-SMAD, was dramatically increased in the ablated hearts compared with the control hearts at 5 dpf/2 dpt (Figures 1H,I, 78.0 ± 8.6 vs. 9.1 ± 1.4 per heart, *N* = 11 and 8, respectively). The pSmad3-positive cells were not restricted in the myocardium only but also located in the epicardium and the outflow tract (OFT). Overall, these results indicated that the TGF-β/Smad3 signaling pathway was activated during zebrafish ventricular regeneration.

### Inhibition of the TGF-β/Smad3 Signaling Pathway Impedes Ventricular Regeneration

**FIGURE 2:**
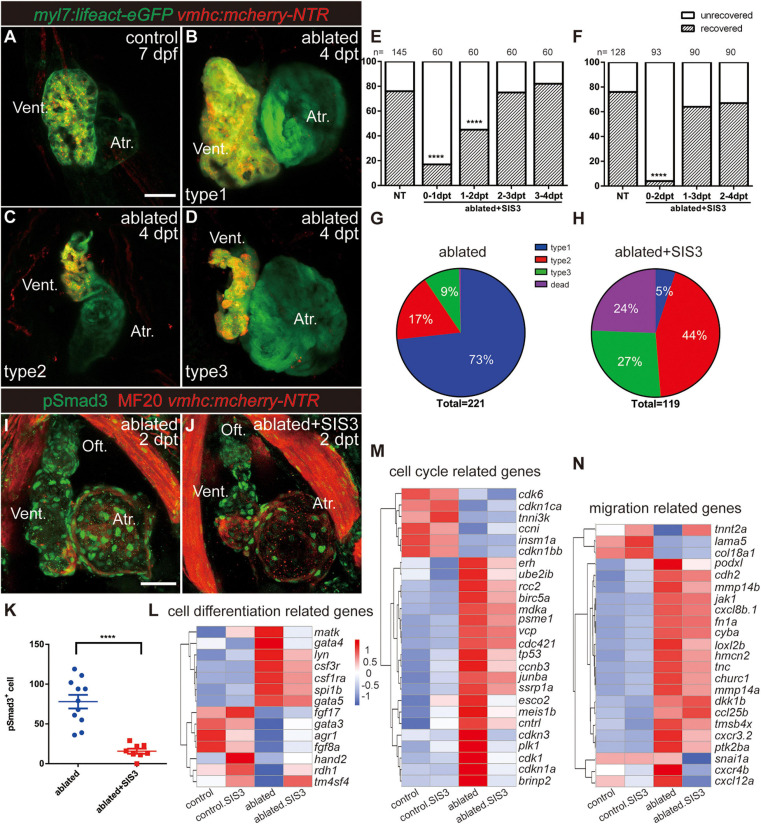
Inhibition of TGF-β/Smad3 signaling pathway impedes ventricular regeneration. **(A–D)** Representative fluorescence images showing the morphology of control *Tg(myl7:lifeact-eGFP; vmhc:mCherry-NTR)* hearts **(A)** and three types of ablated hearts at 7 dpf/4 dpt, fully regenerated ventricle (**B**, type 1), tiny ventricle (**C**, type 2), and partially regenerated ventricle (**D**, type 3). **(E,F)** Quantification of the heart regeneration ratio at 7 dpf/4 dpt in the ablated larvae with different length of SIS3 treatment. The numbers of larvae analyzed for each condition are indicated above the chart. Chi-square test, ****P < 0.0001. **(G,H)** Pie charts show cardiac morphology classification in the ablated larvae without **(G)** or with a 48-h SIS3 treatment **(H)** at 4 dpt. *N* = 221 and 119, respectively. **(I,J)** Representative immunostaining images of *Tg(vmhc:mCherry-NTR)* hearts showing that phospho-Smad3 signal was decreased in the ablated hearts at 5 dpf/2 dpt upon a 48-h SIS3 treatment **(J)**. Green, anti-pSmad3; red, MF20 (anti-MHC). **(K)** Quantification of phospho-Smad3-positive cells in the ablated hearts without or with a 48-h SIS3 treatment at 5 dpf/2 dpt (*N* = 11 and 8, respectively). Mean ± s.e.m., Student’s *t*-test, two-tailed, ****P < 0.0001. **(L–N)** Transcriptomic analysis revealed differentially expressed genes involved in cell differentiation, cell cycle, and migration. Scale bars, 50 μm. dpf, days post-fertilization; dpt, days post-treatment; atr., atrium; oft., out flow tract; vent., ventricle.

After ventricle ablation, most hearts could fully regenerate and contract normally like the control hearts at 7 dpf/4 dpt (Figures 2A,B, type 1). The unrecovered hearts could be classified into two categories based on their cardiac morphology: one category included hearts with tiny shrunken or elongated ventricles (Figure 2C, type 2), and one category included hearts with partially regenerated ventricles (Figure 2D, type 3), presumably due to failure in CM proliferation and/or migration. The percentages of the different types of regenerated hearts at 7 dpf/4 dpt were 73, 17, and 9%, and 1% of the larvae died (Figure 2G, *N* = 221).

To investigate the role of the TGF-β signaling pathway in cardiac regeneration, we acquired a *tgfb1a*^–/–^ mutant line (Xing et al., 2015) and generated a *tgfb1b*^–/–^ mutant line via CRISPR/Cas9 technique. The *tgfb1b*^–/–^ mutant possessed a 5-bp deletion in the first exon of genomic DNA, which presumably resulted in a truncated peptide product of 62 aa instead of the full-length protein of 379 aa (Supplementary Figures 1A,B). The cardiac development and morphology appeared normal in both *tgfb1a*^–/–^ and *tgfb1b*^–/–^ single mutants, and mutation of either *tgfb1* homolog seemed to have no inhibitory effect on the recovery of the injured ventricles (Supplementary Figures 1C–F). Homozygous *tgfb1a*^–/–^; *tgfb1b*^–/–^ mutants were indistinguishable from their siblings in embryonic stages, but we failed to acquire adult homozygous double mutants. Careful examination revealed a subset of juvenile fish with shorter body length and lower body weight in a population of mixed genotypes (Supplementary Figures 2A–F). These fish were prone to death and confirmed as homozygous *tgfb1a*^–/–^; *tgfb1b*^–/–^ mutants via genotyping using tailfin genomic DNA (Supplementary Figure 2G). Therefore, we used several pharmacological inhibitors targeting different components of TGF-β signaling, including SIS3, SB431542, and LY364947 (Sethi et al., 2011), to examine the function of TGF-β signaling in ventricular regeneration. All three inhibitors dramatically reduced the percentages of recovered hearts at 7 dpf/4 dpt (Supplementary Figure 3A).

Next, we focused on the effect of SIS3, a selective Smad3 inhibitor that attenuates Tgfβ1-dependent Smad3 phosphorylation and DNA binding (Jinnin et al., 2006; Denis et al., 2016). A 24-h treatment scheme revealed that SIS3 treatment at 0–1 and 1–2 dpt significantly reduced the ventricle recovery ratio from 76 to 17% and 45% at 7 dpf/4 dpt, respectively, while SIS3 treatment at 2–3 and 3–4 dpt had no obvious effect (Figure 2E). The most dramatic impact was observed after the 48-h SIS3 treatment at 0–2 dpt, in which the recovery ratio decreased to 5% (Figure 2F). Forty-four percent of the larvae showed tiny ventricles, 27% had partially regenerated ventricles, and the other 24% died (Figure 2H and Supplementary Figure 4). Immunofluorescence staining of phospho-Smad3 and phospho-Smad1/5/9 was performed to confirm the specificity of the SIS3 treatment. Although the numbers of both pSmad3-positive cells and pSmad1/5/9-positive cells increased in the myocardium, epicardium, and OFT of regenerating hearts after ventricle ablation, SIS3 specifically inhibited the phosphorylation of Smad3 but not the phosphorylation of Smad1/5/9 (Figures 2I–K and Supplementary Figures 5, 6).

To explore the function of Smad3 in ventricular regeneration, we carried out transcriptomic analysis using four pools of approximately 600, 800, 1,200, and 6,500 manually dissected hearts from the control, SIS3-treated control, ablated, and SIS3-treated ablated groups, considering that fewer CMs remained in the SIS3-treated ablated hearts. Differentially expressed genes were enriched with gene ontology (GO) terms associated with cell differentiation [*gata4* (Denis et al., 2016)], cell proliferation [*plk1* (Jopling et al., 2010), *tnni3k* (Patterson et al., 2017), and *erh* (Weng and Luo, 2013)], and cell migration in wound healing [*tmsb4x* (Bock-Marquette et al., 2004) *and fn* (Trinh and Stainier, 2004; Valiente-Alandi et al., 2018)] (Figures 2L–N). Taken together, our results suggest that the TGF-β/Smad3 signaling pathway is required for ventricular regeneration and may be involved in multiple processes during regeneration.

### Smad3 Inhibition Has Limited Effects on Sarcomere Disassembly and Cardiogenic Factor Reactivation During Regeneration

**FIGURE 3:**
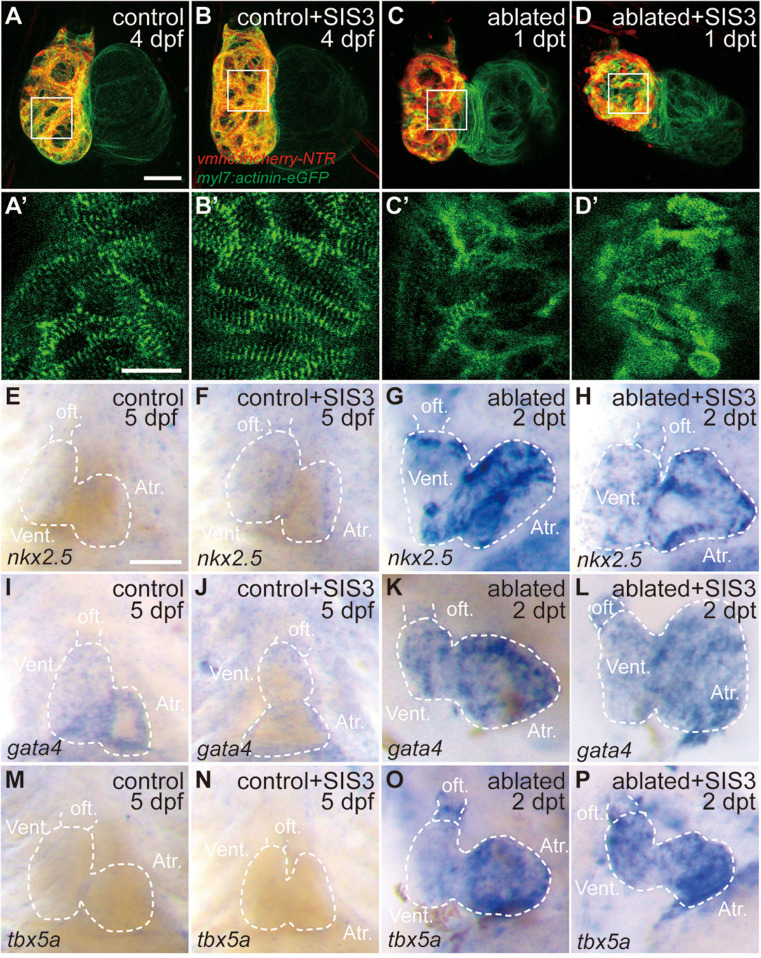
Smad3 inhibition has limited effects on sarcomere disassembly and cardiogenic factor reactivation during regeneration. **(A–D′)** Representative fluorescence images of *Tg(vmhc:mCherry-NTR; myl7:actinin-eGFP)* hearts at 4 dpf/1 dpt indicate that sarcomere disassembly occurred in the ablated hearts **(C,C′)** and SIS3-treated ablated hearts **(D,D′)**. **(A′–D′)** Enlargement of box areas in panels **(A–D)**, green channel only. **(E–P)** Whole-mount *in situ* hybridization showing the expression level changes of cardiogenic factors *nkx2.5*, *gata4*, and *tbx5a* during regeneration. Smad3 inhibition via SIS3 treatment could abolish the reactivation of *nkx2.5* at 5 dpf/2 dpt **(G,H)** but has no effect on the reactivation of *gata4* and *tbx5a* **(K,L,O,P)**. Dashed lines outline the hearts. Scale bars **(A–D,E–P)** 50 μm and **(A′–D′)** 20 μm. dpf, days post-fertilization; dpt, days post-treatment; atr., atrium; oft., out flow tract; vent., ventricle.

Previous reports showed sarcomere disassembly occurred in the injured hearts, which allowed CM to proliferate (Jopling et al., 2010; Zhang et al., 2013). To examine the effect of Smad3 inhibition on sarcomere structure, we used *Tg(myl7:actinin-EGFP)* transgenic fish to observe the morphological changes in sarcomeres during ventricle regeneration (Figures 3A–D′). While the control and SIS3-treated control hearts displayed intact striated sarcomeres, SIS3-treated ablated hearts showed a pattern of sarcomere disarray similar to that in the ablated hearts at 4 dpf/1 dpt. These results suggested that Smad3 inhibition did not affect cardiac regeneration by blocking sarcomere disassembly.

Cardiac regeneration was also accompanied by reactivation of cardiogenic genes. WISH showed that the expressions of cardiogenic transcription factors, such as *nkx2.5*, *gata4*, and *tbx5a*, were significantly increased in the ablated hearts. Smad3 inhibition via SIS3 treatment abolished the reactivation of *nkx2.5* at 5 dpf/2 dpt; however, it had no obvious effect on the reactivation of *gata4* and *tbx5a* (Figures 3E–P). These results suggested that Smad3 inhibition had a limited effect on cardiogenic factor reactivation during ventricle regeneration.

### Smad3 Inhibition Reduces Cardiomyocyte Proliferation Through Cell Cycle Arrest During Regeneration

**FIGURE 4:**
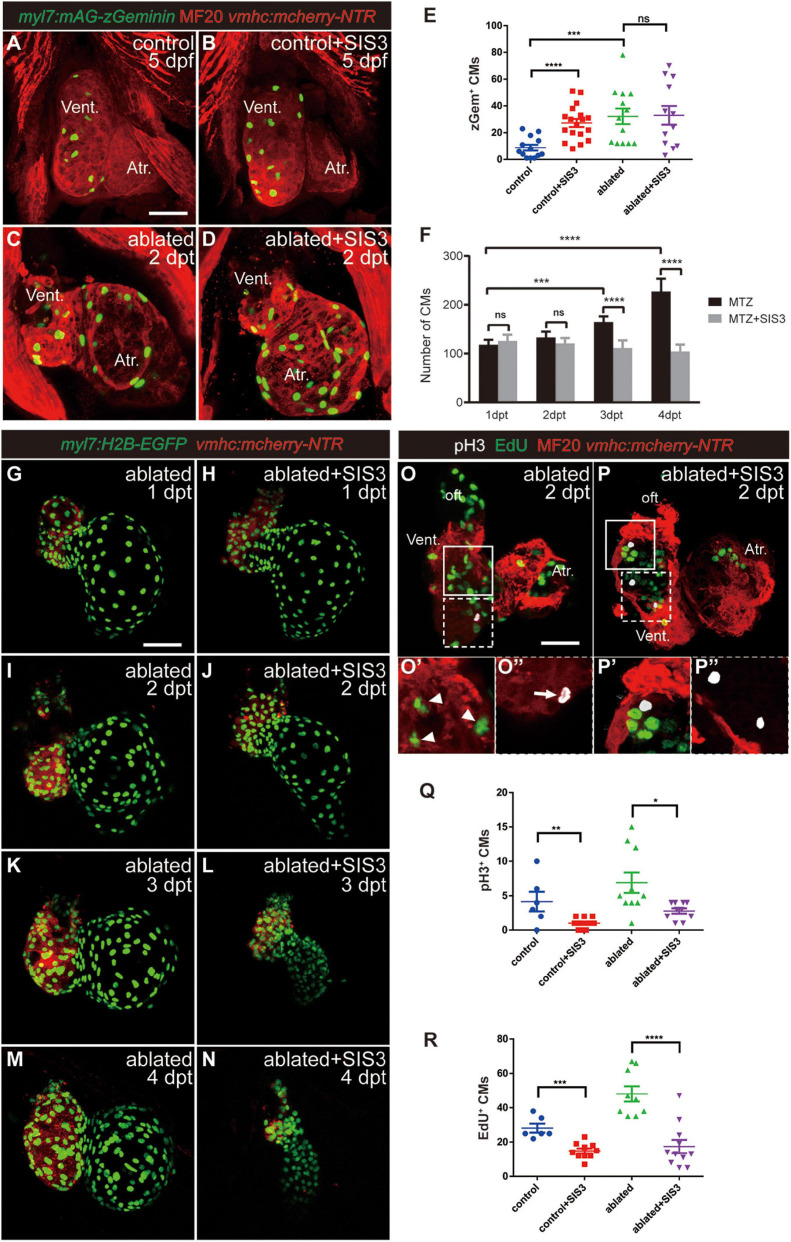
Smad3 inhibition affects CM proliferation during regeneration. **(A–D)** Representative fluorescence images of *Tg(vmhc:mCherry-NTR; myl7:mAG-zGeminin)* larvae with immunostaining of MF20 (anti-MHC) at 5 dpf/2 dpt showing zGeminin-positive CMs in the control or ablated hearts without or with SIS3 treatment. **(E)** Quantification of zGeminin-positive CM number in the control or ablated hearts without or with SIS3 treatment at 5 dpf/2 dpt. *N* = 13, 18, 13, and 12, respectively. Mean ± s.e.m., Student’s *t*-test, two-tailed; ns, non-significant; ***P < 0.001, ****P < 0.0001. **(F)** Quantification of CM nucleus number in the ablated hearts without or with SIS3 treatment at various time points. *N* = 8 for each group. Mean ± s.e.m., Student’s *t*-test, two-tailed; ns, non-significant; ***P < 0.001, ****P < 0.0001. **(G–N)** Representative fluorescence images of *Tg(vmhc:mCherry-NTR; myl7:H2B-EGFP)* ablated hearts without or with SIS3 treatment at various time points showing the change of CM nucleus number. **(O–P″)** Representative fluorescent images of *Tg(vmhc:mCherry-NTR)* hearts with immunostaining of phospho-histone H3 (white), MF20 (red), and EdU (green) in the ablated hearts without or with SIS3 treatment at 5 dpf/2 dpt. **(O,P)** Maximal projections of z-stack images, overlay of three channels. **(O′,P′)** Optical sections of enlargement of box area in panels **(O,P)**, overlay of red and green channels. **(O″,P″)** Optical sections of enlargement of dashed box area in panels **(O,P)**, overlay of red and white channels. Arrowheads point to EdU^+^ CMs; arrow points to pH3^+^ CMs. **(Q)** Quantification of pH3-positive CM number in the control or ablated hearts without or with SIS3 treatment at 5 dpf/2 dpt. *N* = 6, 11, 10, and 10, respectively. Mean ± s.e.m., Student’s *t*-test, two-tailed, *P < 0.05, **P < 0.01. **(R)** Quantification of EdU-positive CM number in the control or ablated hearts without or with SIS3 treatment at 5 dpf/2 dpt. *N* = 6, 10, 9, and 11, respectively. Mean ± s.e.m., Student’s *t*-test, two-tailed, ***P < 0.001, ****P < 0.0001. Scale bars, 50 μm. dpf, days post-fertilization, dpt, days post-treatment; atr., atrium; oft., out flow tract; vent., ventricle; CM, cardiomyocyte.

To observe CM proliferation in real time during ventricle regeneration, we acquired the transgenic line *Tg(myl7:mAG-zGeminin)*, which uses fluorescent ubiquitylation-based cell cycle indicator (FUCCI) technology (Sakaue-Sawano et al., 2008) that can label CMs in the S/G2/M phases with green fluorescence (Figures 4A–E and Supplementary Figure 7). zGem^+^ CMs could be observed in the control hearts at 4 and 5 dpf, mainly in the ventricle due to trabecular initiation and growth at this time point. After ventricle ablation, the number of zGem^+^ CMs in the ablated hearts, in both the ventricle and atrium, was dramatically increased compared with that in the control hearts (35.1 ± 3.9 vs. 20.7 ± 2.8 at 4 dpf/1 dpt, *N* = 14 and 12, respectively; 32.2 ± 5.8 vs. 8.9 ± 2.2 at 5 dpf/2 dpt, *N* = 13 and 13, respectively). Surprisingly, Smad3 inhibition did not significantly reduce the number of zGem^+^ CMs in the SIS3-treated ablated hearts (28.6 ± 4.9 at 4 dpf/1 dpt, *N* = 11; 33.0 ± 6.9 at 5 dpf/2 dpt, *N* = 12), which is contradictory to a previous report that Smad3 inhibition impaired CM proliferation (Dogra et al., 2017). Our results also showed that SB431542 treatment reduced EdU incorporation in CMs at 5 dpf/2 dpt (Supplementary Figures 3B–D). Thus, we examined the change of CM number during regeneration with or without SIS3 treatment using *Tg(myl7:H2B-EGFP)* transgenic fish, which specifically labeled CM nucleus (Figures 4F–N and Supplementary Movies 1–4). The CM numbers were similar in ablated and SIS3-treated ablated groups at the beginning (118.3 ± 9.9 vs. 125.9 ± 12.6 at 4 dpf/1 dpt, 133.1 ± 11.6 vs. 120.8 ± 12.8 at 5 dpf/2 dpt, *N* = 8 for each group). However, the CM number increased in ablated group along with progression of regeneration, but it remained unchanged or even declined in SIS3-treated ablated group with statistically significant differences (165.1 ± 11.1 vs. 111.4 ± 15.5 at 6 dpf/3 dpt, 227.3 ± 25.9 vs. 104.4 ± 13.8 at 7 dpf/4 dpt, *N* = 8 for each group).

To resolve this discrepancy, we examined CM proliferation after Smad3 inhibition during ventricular regeneration using traditional phospho-histone H3 (pH3) immunostaining as well as pulsed EdU incubation and staining (Figures 4O–R). The results showed that the number of pH3^+^ CMs (Figure 4O′, arrowheads) was increased in the ablated hearts (6.9 ± 1.5, *N* = 10) compared with the control hearts (4.2 ± 1.4, *N* = 6). Upon SIS3 treatment, although there were still strong pH3 signals in the non-CMs (Figure 4P), the number of pH3^+^ CMs decreased in the SIS3-treated control hearts (1.0 ± 0.2, *N* = 11) and SIS3-treated ablated hearts (2.8 ± 0.4, *N* = 10) (Figure 4Q). EdU staining exhibited a similar pattern (Figure 4O″, arrow). SIS3 treatment reduced the number of EdU^+^ CMs in both the control hearts (28.2 ± 2.6 vs. 14.9 ± 1.4, *N* = 6 and 10, respectively) and the ablated hearts (48.1 ± 4.5 vs. 17.5 ± 3.9, *N* = 9 and 11, respectively) (Figure 4R), which was consistent with a previous report (Dogra et al., 2017).

We noticed that the number of zGem^+^ CMs was slightly lower, though not statistically significantly lower, in the control hearts treated with SIS3 for 24 h than in those not treated with SIS3 at 4 dpf (Supplementary Figure 7E, 13.7 ± 1.7 vs. 20.7 ± 2.8, *N* = 9 and 12, respectively). However, when the SIS3 treatment was extended to 48 h, the number of zGem^+^ CMs in the control hearts dramatically increased at 5 dpf (Figure 4E, 27.3 ± 3.0 vs. 8.9 ± 2.2, *N* = 18 and 13, respectively). zGem^+^ cells are in the middle-to-late S phase, G2 phase, and early-to-middle M phase of the cell cycle, while transient EdU labeling and pH3 staining indicate cells in the S and M phases, respectively (Choi et al., 2013). Thus, we speculated that SIS3 treatment may cause cell cycle arrest at the G2 phase. We co-stained *Tg(myl7:mAG-zGeminin)* for EdU and pH3 simultaneously (Supplementary Figure 8) and subtracted the numbers of zGem^+^/EdU^+^ CMs and zGem^+^/pH3^+^ CMs from the total number of zGem^+^ CMs in the same heart. The results revealed that the number of zGem^+^/pH3^–^/EdU^–^ CMs was significantly increased in the control hearts after SIS3 treatment at 5 dpf (51.5 ± 5.7 vs. 23.7 ± 3.3, *N* = 13 and 11, respectively), suggesting that Smad3 inhibition via SIS3 treatment may block CM proliferation through cell cycle arrest.

### Smad3 Inhibition Impairs Cardiomyocyte Migration by Weakening the Epithelial–Mesenchymal Transition-Like Response During Regeneration

**FIGURE 5:**
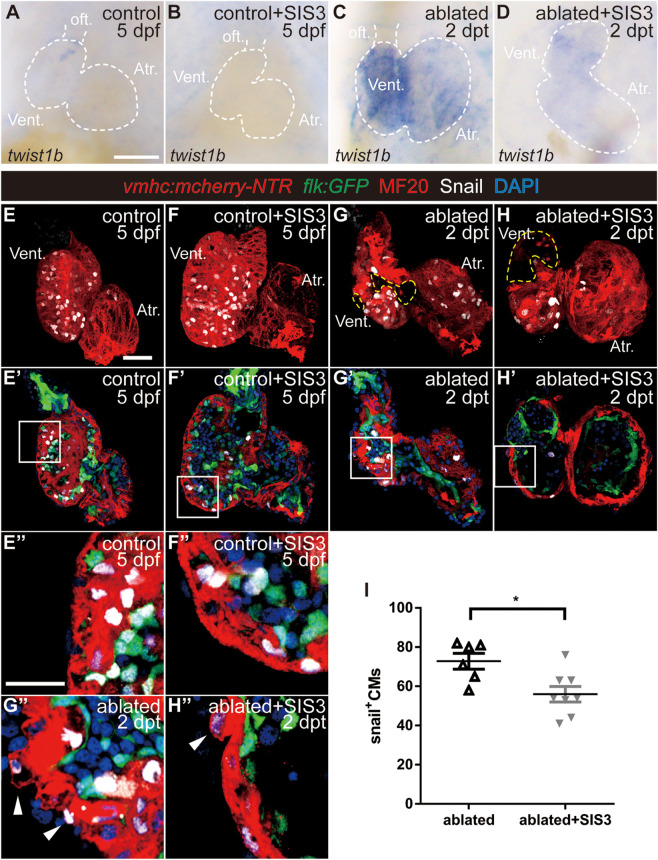
Smad3 inhibition reduces *twist* expression and Snail-positive CM number during regeneration. **(A–D)** Whole-mount *in situ* hybridization showing the expression level change of the EMT marker *twist1b* during regeneration. Smad3 inhibition via SIS3 treatment reduced the upregulation of *twist1b* at 5 dpf/2 dpt. Dashed lines outline the hearts. **(E–H″)** Representative fluorescent images of *Tg(vmhc:mCherry-NTR; flk:GFP)* hearts with immunostaining of Snail (white), MF20 (red), and DAPI (blue) in the control or ablated hearts without or with SIS3 treatment at 5 dpf/2 dpt. **(E–H)** Maximal projections of z-stack images, overlay of red and white channels; yellow dashed lines outline the ablated area. **(E′–H′)** Optical sections of panels **(E–H)**, overlay of four channels. **(E″–H″)** Enlargement of box area in panels **(E′–H′)**; arrowheads point to extruding Snail^+^ CMs into the outer layer. **(I)** Quantification of Snail^+^ CM number in the ablated hearts without or with SIS3 treatment at 5 dpf/2 dpt. *N* = 6 and 8, respectively. Mean ± s.e.m., Student’s *t*-test, two-tailed, *P < 0.05. Scale bars, **(A–D)** 50 μm, **(E–H′)** 20 μm, and **(E″–H″)** 10 μm. dpf, days post-fertilization, dpt, days post-treatment; atr., atrium; oft., out flow tract; vent., ventricle; CM, cardiomyocyte; EMT, epithelial–mesenchymal transition.

It has been reported that CM migration occurs during cardiac regeneration (Itou et al., 2012; Tahara et al., 2016) and that CMs undergo EMT-like response in a mouse cardiac infarction model (Aharonov et al., 2020). Thus, it is of great interest to explore if CMs acquire EMT-like response during ventricle regeneration as well as the roles of the TGF-β/Smad3 signaling pathway in this process. We first examined the expression of the EMT marker Twist1b, a transcription factor that plays an essential role in metastasis (Yang et al., 2004). The average fluorescence intensity of the Twist1b protein in the ablated hearts was much higher than that in the control hearts (Supplementary Figure 9). WISH showed that *twist1b* gene expression was upregulated in the ablated hearts compared with the control hearts and that SIS3 treatment abolished this upregulation (Figures 5A–D).

Next, we examined the expression of Snail, another transcription factor and EMT marker (Wu and Zhou, 2010). Immunostaining showed that Snail^+^ CMs were uniformly distributed in the ventricles of the control hearts (Figure 5E and Supplementary Movie 5) and SIS3-treated control hearts (Figure 5F and Supplementary Movie 6). In the ablated hearts, Snail^+^ CMs accumulated around the lost area in the ventricle and scattered throughout the atrium (Figure 5G and Supplementary Movie 7). Further analysis demonstrated that Snail^+^ CMs were distributed in both the ventricular compact layer and trabecular layer in the control hearts (Figures 4E′,E″), which was consistent with trabecular formation at this stage. In the ablated hearts, the number of Snail^+^ CMs in the trabecular layer decreased, while the number in the compact layer and atrium slightly increased (Supplementary Figure 10), and Snail^+^ CMs frequently extruded from the myocardium into the outer layer (Figures 4G′,G″, arrowheads). Smad3 inhibition via SIS3 treatment reduced the number of Snail^+^ CMs in the whole ablated hearts at 5 dpf (Figure 5I, 56.0 ± 4.0 vs. 72.8 ± 4.0, *N* = 8 and 6, respectively), and Snail^+^ CMs often located away from the lost area (Figure 5H and Supplementary Movie 8). Snail^+^ CM number reduction was also observed after SB431542 treatment (Supplementary Figures 3E–G).

**FIGURE 6:**
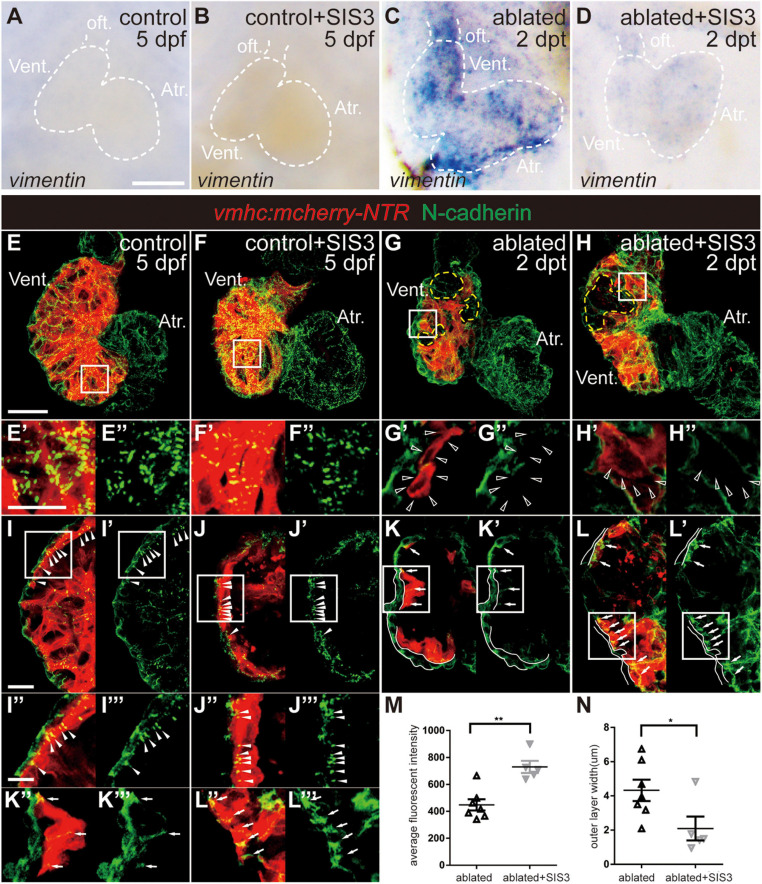
Smad3 inhibition affects *vimentin* and N-cadherin expression during regeneration. **(A–D)** Whole-mount *in situ* hybridization showing the expression level change of *vimentin* during regeneration. Smad3 inhibition via SIS3 treatment reduced the upregulation of *vimentin* at 5 dpf/2 dpt. Dashed lines outline the hearts. **(E–H″)** Maximal projection images of *Tg(vmhc:mCherry-NTR)* hearts showing surface view of N-cadherin immunostaining (green) in the control or ablated hearts without or with SIS3 treatment at 5 dpf/2 dpt. **(E′–H′)** Enlargement of box area in panels **(E–H)**. **(E″–H″)** Green channel only. Yellow dashed lines outline the ablated areas; open arrowheads point to N-cadherin-absent CMs adjacent to the ablated areas. **(I–L″′)** Optical section images of *Tg(vmhc:mCherry-NTR)* hearts showing the cross-section view of N-cadherin immunostaining (green) in the ventricle. **(I″–L″)** Enlargement of box area in panels **(I–L)**. **(I′–L′,I″′–L″′)** Green channel only. Arrowheads point to regular punctate N-cadherin distribution; arrows point to irregularly dispersed N-cadherin distribution; white lines outline the outer layer without CM marker. **(M)** Quantification of average fluorescence intensity of N-cadherin immunostaining in the ablated hearts without or with SIS3 treatment at 5 dpf/2 dpt. *N* = 7 and 5, respectively. Mean ± s.e.m., Student’s *t*-test, two-tailed, **P < 0.01. **(N)** Quantification of the outer layer width of N-cadherin immunostaining in the ablated hearts without or with SIS3 treatment at 5 dpf/2 dpt. *N* = 7 and 5, respectively. Mean ± s.e.m., Student’s *t*-test, two-tailed, *P < 0.05. Scale bars, **(A–D)** 50 μm, **(E–H)** 20 μm, **(E′–H″,I″–L″′)** 5 μm, **(I–L′)** 10 μm. dpf, days post-fertilization, dpt, days post-treatment; atr., atrium; oft., out flow tract; vent., ventricle; CM, cardiomyocyte.

We also examined the changes in the expression of other EMT markers during ventricle regeneration after Smad3 inhibition. WISH of *vimentin*, a gene encoding an intermediate filament protein that is important for cell migration, revealed that its upregulation in the ablated hearts could also be abolished by SIS3 treatment (Figures 6A–D). Immunostaining of N-cadherin (Figures 6E–N), a cellular adhesion molecule that is vital for cell contact and migration, demonstrated regular punctate N-cadherin distribution along the lateral side of CMs in the trabecular and compact layers in the control hearts (Figures 6E–E″,I–I″′, arrowheads). In the ablated hearts, the N-cadherin signal was absent in CMs adjacent to the ablated area (Figures 6G–G″, open arrowheads) or displayed an irregularly dispersed distribution in other areas (Figures 6K–K″′, arrows). Interestingly, we observed a much thicker outer layer with N-cadherin signal but no myocardial markers in the ablated hearts than in the control hearts, and the identity and physiological function of this layer remain to be explored. SIS3 treatment did not globally affect the distribution of N-cadherin (Figures 6H–H″,L–L″′), although the average fluorescence intensity of N-cadherin in the SIS3-treated ablated hearts increased (Figure 6M). The thickness of the outer layer was also reduced in the ablated hearts after SIS3 treatment (Figure 6N). Taken together, our results indicated that CMs possessed EMT-like response during cardiac regeneration and that Smad3 inhibition via SIS3 treatment weakened the EMT-like response, which may lead to CM migration defect.

## Discussion

The TGF-β/Smad3 signaling pathway has been reported to be vital for scar resolution in an adult zebrafish model of heart cryoinjury (Chablais and Jazwinska, 2012), and our results indicated that this pathway was activated after ventricle ablation and played important roles in ventricle regeneration. The exacerbation of different types of unrecovered hearts after SIS3 treatment and the transcriptomic analysis suggested that TGF-β/Smad3 signaling may affect CM proliferation and migration. Further examination revealed that Smad3 inhibition caused cell cycle arrest and weakened the EMT-like response during regeneration. However, since the control hearts still develop and undergo trabeculation at this stage, we have interpreted the data with extra caution by comparing different compartments of the heart including the ventricular trabecula, compact layer, and atrium.

### The Reactivation of *nkx2.5* Was Repressed by Smad3 Inhibition

During cardiac regeneration, cardiogenic transcription factors, such as *nkx2.5*, *gata4*, and *tbx5a*, are reactivated (Zhang et al., 2013); and inhibition of the Notch signaling pathway abolishes the reactivation of these factors (Li et al., 2020). However, our study revealed that Smad3 inhibition via SIS3 treatment only repressed the expression of *nkx2.5* but not the expression of other cardiogenic transcription factors, suggesting a more specific function of the TGF-β/Smad3 signaling pathway in ventricle regeneration. It has been reported that inhibition of the TGF-β signaling pathway by A83-01 promotes the proliferation and differentiation of *nkx2.5*^+^ CMs and improves cardiac function after MI (Chen and Wu, 2012; Chen et al., 2015). A83-01 inhibits the ALK5, ALK4, and ALK7 receptors, blocking the phosphorylation and activation of Smad2. In contrast, SIS3 attenuates TGFβ1-dependent Smad3 phosphorylation but has no effect on Smad2 (Jinnin et al., 2006; Denis et al., 2016). Considering the opposing roles of Smad2 and Smad3 in CM proliferation during development (Dogra et al., 2017), we speculate that Smad2 and Smad3 also have opposing functions in *nkx2.5* reactivation and CM proliferation during ventricle regeneration, which warrants further investigation if specific Smad2 antibodies become available.

### Cardiomyocyte Cell Cycle Arrest by Smad3 Inhibition

The FUCCI system can reveal cell cycle progression in real time (Sakaue-Sawano et al., 2008); thus, we used the transgenic line *Tg(myl7:mAG-zGeminin)* to label CMs in the S/G2/M phases. CM proliferation in the control hearts was mainly in the ventricle due to trabecular initiation and growth. However, zGem^+^ CMs in the ablated hearts, mainly in the atrium, remained fluorescent after Smad3 inhibition. The number of zGem^+^ CMs was not changed after a 24-h SIS3 treatment but was significantly increased in the 48-h SIS3-treated control hearts at 5 dpf. Through co-staining with pH3 and transient EdU labeling in the same heart, we revealed that the number of zGem^+^/pH3^–^/EdU^–^ CMs increased, suggesting cell cycle arrest at G2 phase. It has been reported that the TGF-β signaling inhibitor SB431542 causes CM cell cycle arrest at the G2/M phase through *p27* inhibition (Yang et al., 2010; Mohamed et al., 2018). We found no difference in the expression of *p27* in the SIS3-treated hearts compared with the control hearts (data not shown). This result may occur due to the low basal expression level of *p27*, making it difficult to detect a reduction after SIS3 treatment.

### Cardiomyocyte Epithelial–Mesenchymal Transition-Like Response Is Reduced by Smad3 Inhibition

Cardiac regeneration requires coordination between CM proliferation and migration, and cardiac regeneration in both mammalian and zebrafish is accompanied by CM migration (Jopling et al., 2010; Kikuchi et al., 2010; Kim et al., 2010; Itou et al., 2012). How the EMT-like process plays a role in CM migration during regeneration remains to be explored. We discovered that the expression of the EMT marker *twist1b* was increased in the ablated hearts compared with the control hearts. CMs that express Snail, which is another EMT marker, were mainly distributed in the trabecular layer due to trabeculation in the control hearts at this stage (Cherian et al., 2016), while in the ablated hearts, Snail^+^ CMs were enriched near the wounded area and scattered in the atrium. Interestingly, a few Snail^+^ CMs extruded from the myocardium into the outer layers, which were multiple cells thick in the ablated hearts, as revealed by N-cadherin and DAPI staining, instead of the single epicardial layer observed in the control hearts. The localization of N-cadherin also changed from a regular punctate distribution on the lateral side of the compact layer in the control hearts to an absent or irregular distribution in the ablated hearts, suggesting that CMs were ready to migrate. Smad3 inhibition abolishes *twist1b* upregulation, reduces Snail^+^ CM numbers, and increases N-cadherin expression, thus weakening EMT-like response during regeneration. It has been reported that after ventricle resection in newt, a subset of CMs with proliferative abilities migrates toward the epicardium and apex through transformation and then embeds into the regeneration-specific matrix to repair the damaged area (Mercer et al., 2013). We hypothesize that during ventricle regeneration in zebrafish, a subset of CMs also undergoes an EMT process and migrates into the extracellular matrix located between the myocardium and epicardium to enter the ablated area. Conventional confocal technique is not able to document this dynamic process *in vivo*; advanced microscopy, such as light-sheet microscopy with low phototoxicity and photobleaching effect for long-term imaging, combined with novel reporter lines is required for further investigation of migration during zebrafish heart regeneration.

In conclusion, we show that the TGF-β/Smad3 signaling pathway participates in CM cell cycle progression and EMT process during ventricle regeneration, and we lay a foundation for the development of novel therapeutic strategies for MI in the future.

## Materials and Methods

### Zebrafish Husbandry

All zebrafish were raised under standard conditions in accordance with institutional and national animal welfare guidelines. The transgenic lines used in this study were as follows: *Tg(vmhc:mCherry-NTR)*, *Tg(flk:GFP)*, *Tg(myl7:actinin-EGFP)*, *Tg(myl7:H2B-EGFP)*, *Tg(myl7:lifeact-EGFP)*, and *Tg(myl7:mAG-zGeminin)*. 1-Phenyl-2-thiourea (PTU; Sigma, P7629) was added at 24 hpf to prevent pigmentation.

### Generation of *tgfb1b*^–/–^ Mutant Zebrafish

*tgfb1b*^–/–^ mutants were generated using CRISPR/Cas9 technique (Chang et al., 2013). sgRNA target sites were identified by online tool CRISPRscan. The sgRNAs were *in vitro* transcribed as previously reported (Li et al., 2020) and co-injected with Cas9 protein (New England Biolabs) into embryos at the one-cell stage. Positive founders were mated with wild-type or *Tg(vmhc:mCherry-NTR)* fish to obtain F1 generation. The F1 heterozygous zebrafish with identical frameshift mutations were intercrossed to generate F2 homozygous mutants.

### Chemical Treatment

*Tg(vmhc:mCherry-NTR)* larvae were treated with 5 mM of MTZ (Sigma) in E3 water at 72 hpf for 4 h as previously described (Zhang et al., 2013). After being washed, the larvae were then incubated in 2 μM of SIS3 (Selleck), 25 μM of SB431542 (Tocris), 20 μM of LY364947 (Selleck), or 0.2% dimethyl sulfoxide (DMSO) (Thermo Fisher Scientific) as control from 76 to 124 hpf.

### *In situ* Hybridization

Whole-mount *in situ* hybridization was performed using a modified protocol as previously described (He et al., 2020), including *nkx2.5*, *gata4*, *vimentin*, *tgfb1a*, and *smad3a*. The probes for *tgfb1b*, *alk5a*, *alk5b*, *tgfb2*, *tgfb3*, *twist1b*, and *tbx5a* were amplified by PCR with the following primers: *tgfb1b* F-5′ CC AAGGAACCAGAAGTAGAA, *tgfb1b* R-5′ CCAGACATA GGAGCAAGAG; *alk 5a* F-5′ TCGTGTGCCAAGTGAAGAAG, *alk5a* R-5′ CATGATCTTGGCCATCACAC; *alk5b* F-5′ GG TGTGTGTGCTGTGTTTCC, *alk5b* R-5′ ACTGACGGGTCTG ACTGGAC; *tgfb2* F-5′ ACGCCAAAGAAGTGCACAAG, *tgfb2* R-5′ CGCTCCACAGATACGGACAG; *tgfb3* F-5′ AACCTGA GCACCTCCAGGAC, *tgfb3* R-5′ GCTGCACTTGCAGGAT TTG; *twist1b* F-5′ GAAAACACGAGGACCAATG, *twist1b* R-5′ GAATTGTACTAAAGCTTTGTA. *tbx5a* F-5′ GCGTT TCCAGCACATCTCAG, *tbx5a* R-5′ TGTGTCCAGTGCTCCTT TACC.

### Immunofluorescence

Immunostaining on whole mount larvae or dissected larval hearts was performed as previously described (Yang and Xu, 2012). The primary antibodies used in this study included the following: anti-phospho-Smad3 (rabbit; Abcam, 52903), anti-phospho-Smad1/5/9 (rabbit; CST, 13820), anti-phospho-histone H3 (rabbit; Merck Millipore, 06570), anti-MHC (mouse; DSHB, MF20), anti-Twist (mouse; Santa Cruz, 81417), and anti-Snail (rabbit; Genetex, 125918). The secondary antibodies included the following: Alexa Fluor 488 goat anti-rabbit IgG, Alexa Fluor 488 goat anti-mouse IgG, Alexa Fluor 555 goat anti-mouse IgG, and Alexa Fluor 647 goat anti-rabbit IgG from Thermo Fisher Scientific.

### EdU Treatment

The larvae at 123 hpf were incubated with 500 μM of EdU for 1 h in E3 water containing PTU and 2% DMSO to facilitate EdU solubilization. After treatment, larvae were rinsed with E3 water, anesthetized with 0.2% tricaine, and fixed overnight in 4% paraformaldehyde (PFA). The CLICK-IT reaction for EdU labeling was performed according to the manufacturer’s instruction (Thermo Fisher Scientific).

### Imaging

The larvae and dissected hearts were imaged by LSM710/LSM880 (Zeiss), SP8 (Leica), or A1 (Nikon) confocal microscopes. The acquired confocal z-stack images were processed, and cell counting was performed using ZEN (Zeiss), LAS X (Leica), and Fiji software. The injured area was determined by visually examination of each single optical section and then manually labeled in the maximal projection image.

### RNA Extraction

Zebrafish hearts were manually dissected by tweezers from control and ablated larvae after euthanasia with an overdose of anesthetic (0.2% tricaine). The dissected hearts were washed in 10% fetal bovine serum (FBS) medium and stored in Eppendorf tubes with TRIzol solution (Thermo Fisher Scientific). Different batches were pooled together to reach approximately 600, 800, 1,200, and 6,500 hearts for the control, SIS3-treated control, ablated, and SIS3-treated ablated groups. RNA was extracted using TRIzol solution following manufacturer’s instruction; 1.5 μg of total RNA for each group was sent out for RNA-seq.

### Bioinformatic Analysis

The sequencing data were obtained by RNA-seq conducted by Genewiz company and had been uploaded to Gene Expression Omnibus (GEO) database (accession number GSE162820). The data were monitored by FASTQ quality trimmer by which low-quality reads were removed. High-quality reads were then mapped with the reference genome using HISAT (Kim et al., 2015). The number of reads was counted using HTseq (Anders et al., 2015). DE-seq (Anders and Huber, 2010) was used to analyze the differentially expressed genes. Supervised two-way clustering was conducted using pheatmap package of R software (Diboun et al., 2006).

### Statistical Analysis

The regeneration ratio was calculated as the number of recovered larvae over the number of total injured larvae. GraphPad software was used for statistical analysis. Values were presented as mean ± s.e.m. Statistical significance was defined as ^∗^*P* < 0.05, ^∗∗^*P* < 0.01, ^∗∗∗^*P* < 0.001, and ^****^P < 0.0001, determined by Student’s *t*-test or chi-square test in the quantification of the percentage of recovered hearts.

## Data Availability Statement

The datasets presented in this study can be found in online repositories. The names of the repository/repositories and accession number(s) can be found in the article/Supplementary Material.

## Ethics Statement

The animal study was reviewed and approved by Fudan University Institutional Animal Care and Use Committee.

## Author Contributions

YP and RZ conceived and designed the project and wrote and revised the manuscript. YP, WW, YF, HH, Y-FH, XL, and HL conducted the experiments and analyzed the data. NC, MP, and J-WX provided critical reagents. All authors reviewed the manuscript.

## Conflict of Interest

The authors declare that the research was conducted in the absence of any commercial or financial relationships that could be construed as a potential conflict of interest.

## Supplementary Material

Supplementary Movie 1 3D reconstruction of live imaging of ablated *Tg(vmhc:mCherry-NTR; myl7:H2B-EGFP)* heart at 5 dpf/2 dpt.

Supplementary Movie 2 3D reconstruction of live imaging of SIS3-treated ablated *Tg(vmhc:mCherry-NTR; myl7:H2B-EGFP)* heart at 5 dpf/2 dpt.

Supplementary Movie 3 3D reconstruction of live imaging of ablated *Tg(vmhc:mCherry-NTR; myl7:H2B-EGFP)* heart at 7 dpf/4 dpt.

Supplementary Movie 4 3D reconstruction of live imaging of SIS3-treated ablated *Tg(vmhc:mCherry-NTR; myl7:H2B-EGFP)* heart at 7 dpf/4 dpt.

Supplementary Movie 5 3D reconstruction of fluorescence images of fixed *Tg(vmhc:mCherry-NTR; flk:GFP)* fish with immunostaining of Snail (white) and MF20 (red) in the control heart at 5 dpf/2 dpt.

Supplementary Movie 6 3D reconstruction of fluorescence images of fixed *Tg(vmhc:mCherry-NTR; flk:GFP)* fish with immunostaining of Snail (white) and MF20 (red) in the SIS3-treated control heart at 5 dpf/2 dpt.

Supplementary Movie 7 3D reconstruction of fluorescence images of fixed *Tg(vmhc:mCherry-NTR; flk:GFP)* fish with immunostaining of Snail (white) and MF20 (red) in the ablated hearts at 5 dpf/2 dpt.

Supplementary Movie 8 3D reconstruction of fluorescence images of fixed *Tg(vmhc:mCherry-NTR; flk:GFP)* fish with immunostaining of Snail (white) and MF20 (red) in the SIS3-treated ablated hearts at 5 dpf/2 dpt.
