# Corrigendum: Inhibition of TGF-β/Smad3 Signaling Disrupts Cardiomyocyte Cell Cycle Progression and Epithelial–Mesenchymal Transition-Like Response During Ventricle Regeneration

**DOI:** 10.3389/fcell.2021.699796

**Published:** 2021-05-14

**Authors:** Yuanyuan Peng, Wenyuan Wang, Yunzheng Fang, Haichen Hu, Nannan Chang, Meijun Pang, Ye-Fan Hu, Xueyu Li, Han Long, Jing-Wei Xiong, Ruilin Zhang

**Affiliations:** ^1^School of Life Sciences, Fudan University, Shanghai, China; ^2^Shanghai Medical College, Fudan University, Shanghai, China; ^3^Institute of Molecular Medicine, Beijing Key Laboratory of Cardiometabolic Molecular Medicine, Peking University, Beijing, China; ^4^Department of Medicine, Li Ka Shing Faculty of Medicine, The University of Hong Kong, Pokfulam, China; ^5^School of Biomedical Sciences, Li Ka Shing Faculty of Medicine, The University of Hong Kong, Pokfulam, China; ^6^School of Basic Medical Sciences, Wuhan University, Wuhan, China

**Keywords:** ventricle regeneration, TGF-β, Smad3, cell cycle, EMT-like response

In the original article, there was an error. The correct accession number in GEO database is GSE162820. A correction has been made to *MATERIALS AND METHODS, Bioinformatic Analysis* section. The correct statement should read:

**The sequencing data were obtained by RNA-seq conducted by Genewiz company and had been uploaded to Gene Expression Omnibus (GEO) database (accession number GSE162820)**.

The authors apologize for this error and state that this does not change the scientific conclusions of the article in any way. The original article has been updated.

