# Supplementary material for: Inhibition of TGF-β/Smad3 Signaling Disrupts Cardiomyocyte Cell Cycle Progression and Epithelial–Mesenchymal Transition-Like Response During Ventricle Regeneration
Source: Front Cell Dev Biol. 2021 Mar 16;9:632372. doi: 10.3389/fcell.2021.632372 (PMC8010688; doi:10.3389/fcell.2021.632372)
Supplement: Supplementary file 9 [file Data_Sheet_1.pdf]

Supplementary materials

Supplementary figure 1. Generation of *tgfb1b*$^{-/-}$ mutant fish.

Supplementary figure 2. The morphology and survival curve of *tgfb1a*$^{-/-}$; *tgfb1b*$^{-/-}$ double mutants.

Supplementary figure 3. SB431542 treatment affects cardiomyocyte proliferation and migration.

Supplementary figure 4. Morphology of ablated hearts with SIS3 treatment.

Supplementary figure 5. Dynamic changes of phospho-Smad3 signal during cardiac regeneration.

Supplementary figure 6. SIS3 treatment has no effect on Smad1/5/9 phosphorylation.

Supplementary figure 7. zGeminin positive CM number after 24-hour SIS3 treatment.

Supplementary figure 8. The number of zGem^{+}/pH3^{-}/EdU^{-} CMs is increased after 48-hour SIS3 treatment.

Supplementary figure 9. Twist is upregulated during cardiac regeneration.

Supplementary figure 10. Smad3 inhibition reduces Snail-positive CM number during regeneration.

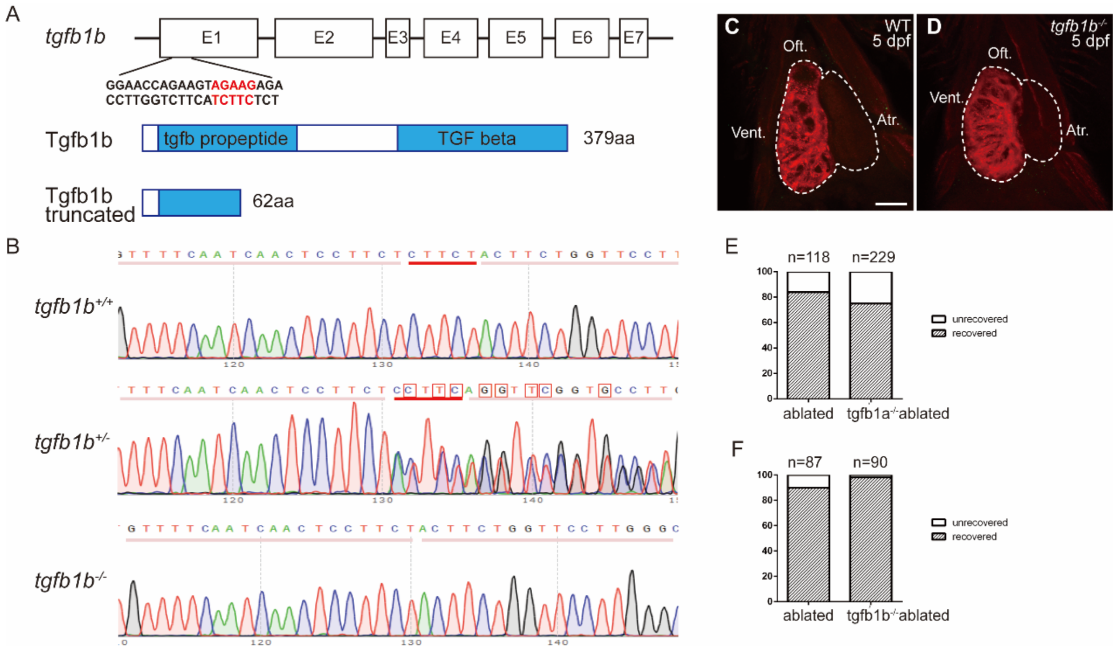

Supplementary figure 1. Generation of *tgfb1b*$^{-/-}$ mutant fish.

(A) Schematic diagram of CRISPR/Cas9 target site in *tgfb1b* locus. 5 bp deletion leads to formation of a premature stop codon which presumably results in a truncated peptide product.

(B) *tgfb1b*$^{-/-}$ genomic editing was confirmed by genotyping.

(C, D) Fluorescence images of *Tg(vmhc:mCherry-NTR)* hearts showed normal cardiac morphology in *tgfb1b*$^{-/-}$ mutants at 5 dpf. Scale bar, 50 µm. dpf, days post fertilization; atr., atrium; oft., outflow tract; vent., ventricle.

(E, F) Quantification of the heart regeneration ratio at 7 dpf/4 dpt in *tgfb1a*$^{-/-}$ mutants and *tgfb1b*$^{-/-}$ mutants. The numbers of larvae analyzed are indicated above the chart.

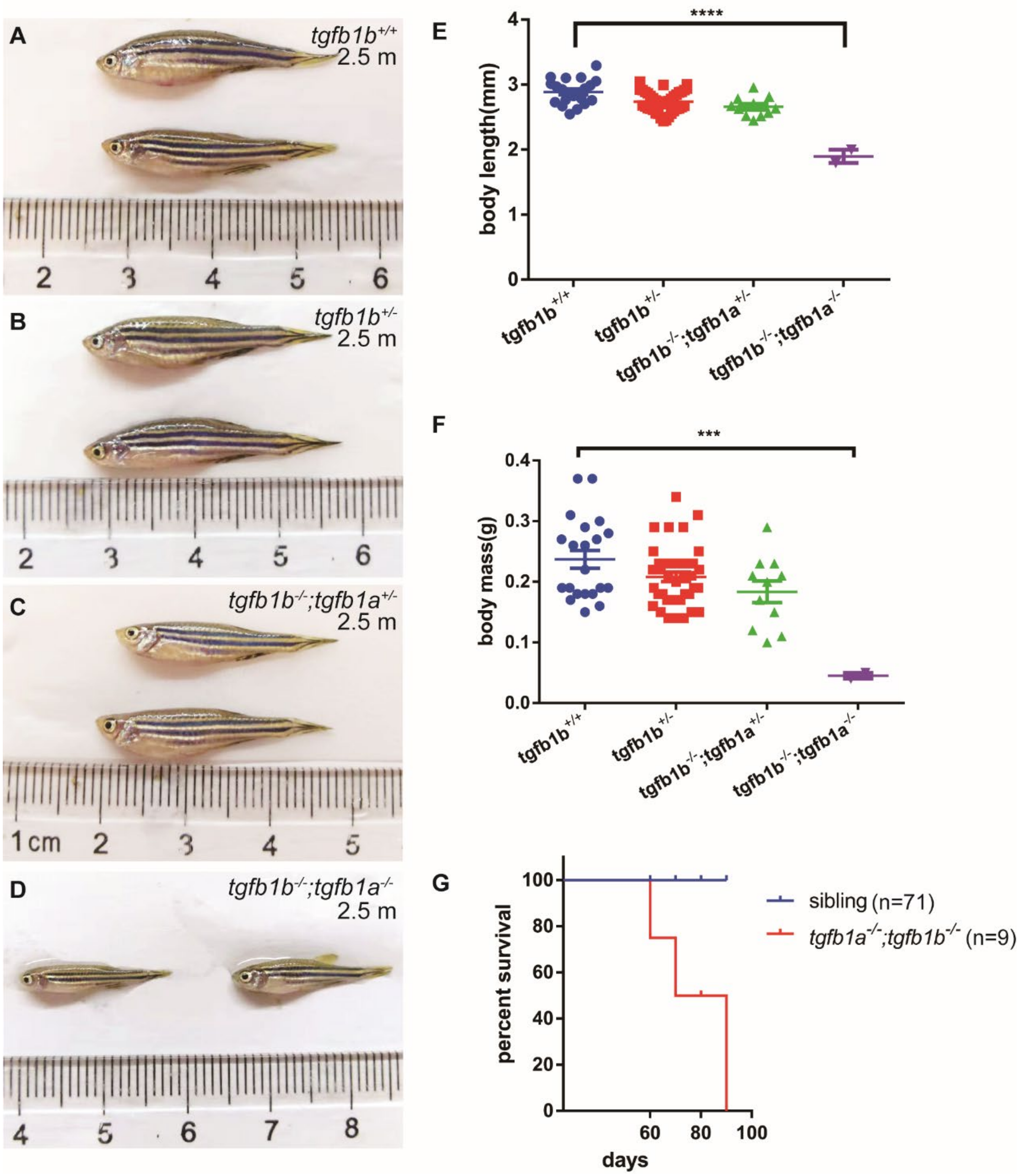

Supplementary figure 2. The morphology and survival curve of *tgfb1a*$^{-/-}$; *tgfb1b*$^{-/-}$ double mutants.

(A-D) The morphology of *tgfb1b*$^{-/-}$; *tgfb1a*$^{-/-}$ double mutants and siblings of different genotypes.

(E, F) Quantification of the body length and body mass in *tgfb1b*$^{-/-}$; *tgfb1a*$^{-/-}$ double mutants and siblings. N=21, 39, 11, 2, respectively. Mean ± s.e.m., Student's t-test, two-tailed, ***, P<0.001, ****, P<0.0001.

(G) Survival curves of *tgfb1b*$^{-/-}$; *tgfb1a*$^{-/-}$ double mutants and siblings. N=9, 71.

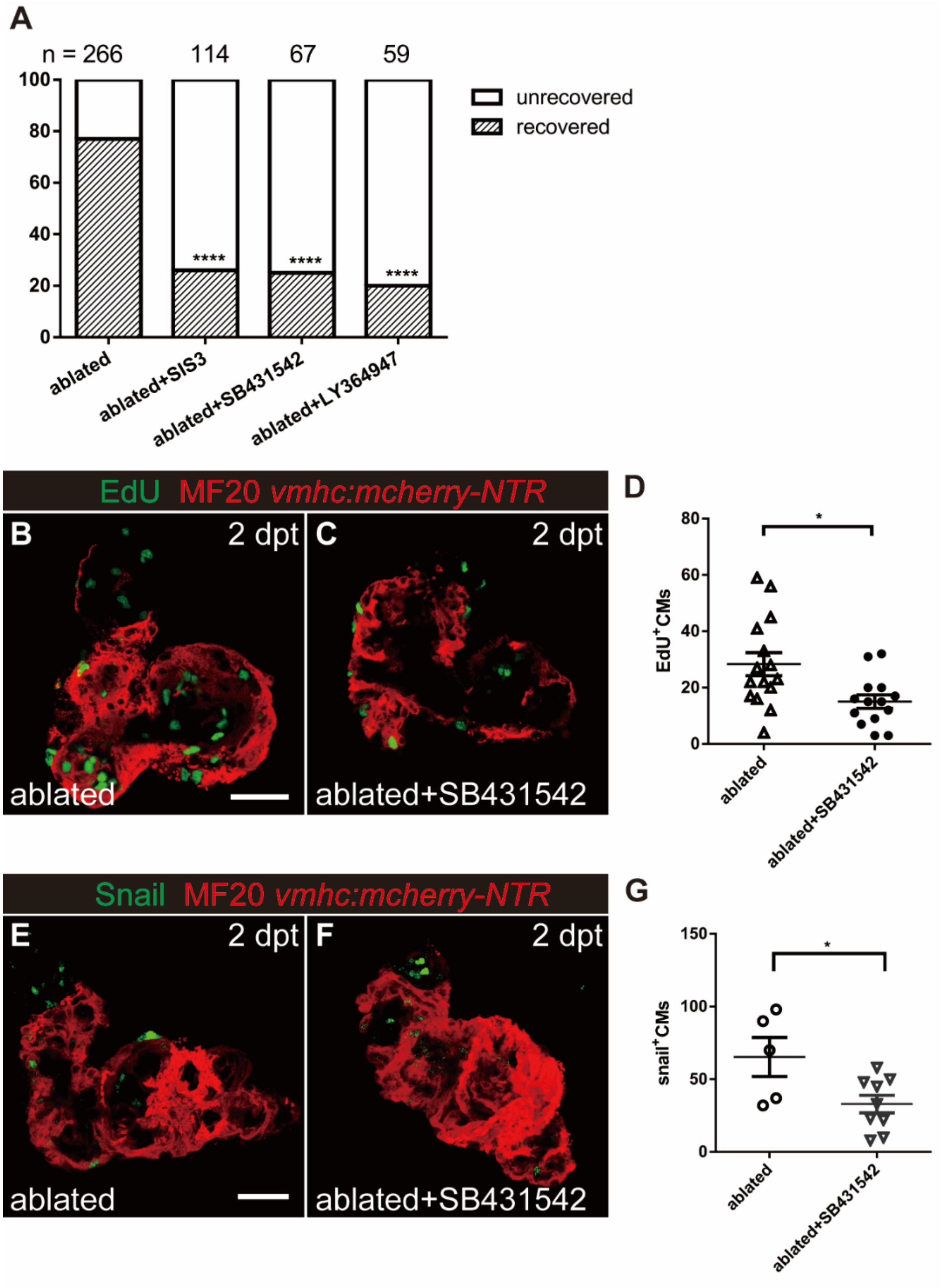

Supplementary figure 3. SB431542 treatment affects cardiomyocyte proliferation and migration.

(A) Quantification of the heart regeneration ratio at 7 dpf/4 dpt in ablated larvae with SIS3, SB431542, LY364947 treatment. The numbers of larvae analyzed for each condition are indicated above the chart. Chi square test, ****, $P<0.0001$.

(B, C) Representative fluorescent images of *Tg(vmhc:mCherry-NTR)* hearts with immunostaining of MF20 (red) and EdU (green) in ablated hearts without or with SB431542 treatment at 5 dpf/2 dpt.

(D) Quantification of EdU positive CM number in ablated hearts without or with SB431542 treatment at 5 dpf/2 dpt. N=15, 14, respectively. Mean ± s.e.m., Student's t-test, two-tailed, *, $P<0.05$.

(E, F) Representative fluorescent images of *Tg(vmhc:mCherry-NTR)* hearts with immunostaining of MF20 (red), Snail (green) in ablated hearts without or with SB431542 treatment at 5 dpf/2 dpt.

(G) Quantification of $Snail^{+}$ CM number in ablated hearts without or with SB431542 treatment at 5 dpf/2 dpt. N=5, 9, respectively. Mean ± s.e.m., Student's t-test, two-tailed, *, $P<0.05$.

Scale bars, 20 μm. dpt, days post treatment.

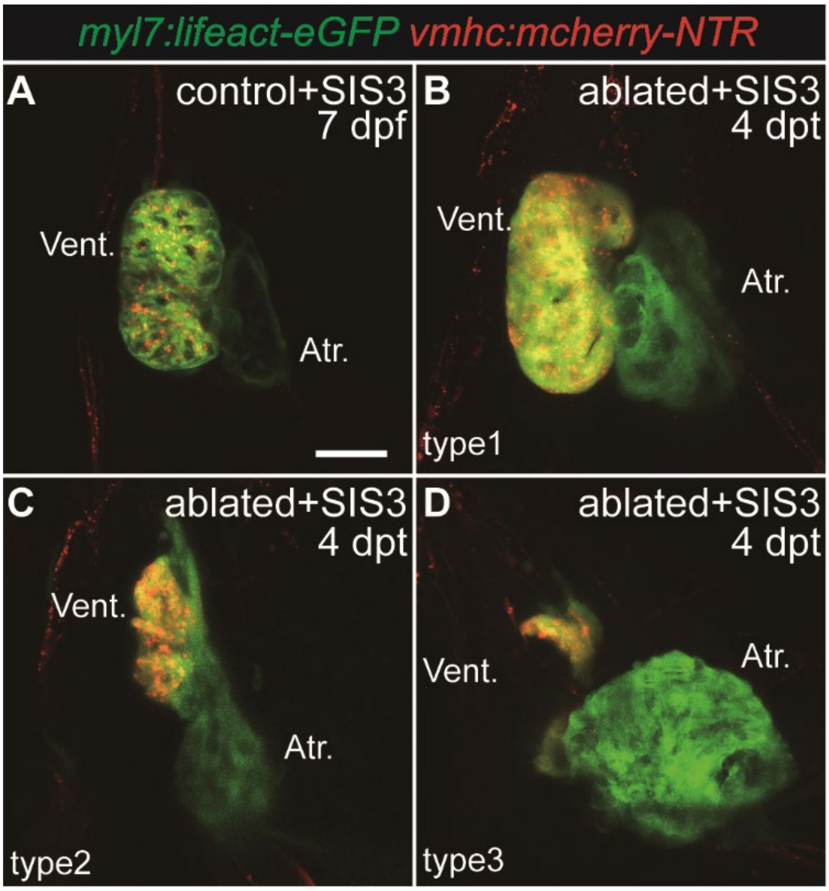

Supplementary figure 4. Morphology of ablated hearts with SIS3 treatment. (A-D) Representative fluorescence images showed the morphology of *Tg(myl7:lifeact-eGFP; vmhc:mCherry-NTR)* control hearts (A) and three types of ablated hearts after SIS3 treatment at 7 dpf/4 dpt, fully-regenerated ventricle (B, type 1), tiny ventricle (C, type 2) and partially-regenerated ventricle (D, type 3). Scale bar, 50 μm. dpt, days post treatment; atr., atrium; vent., ventricle.

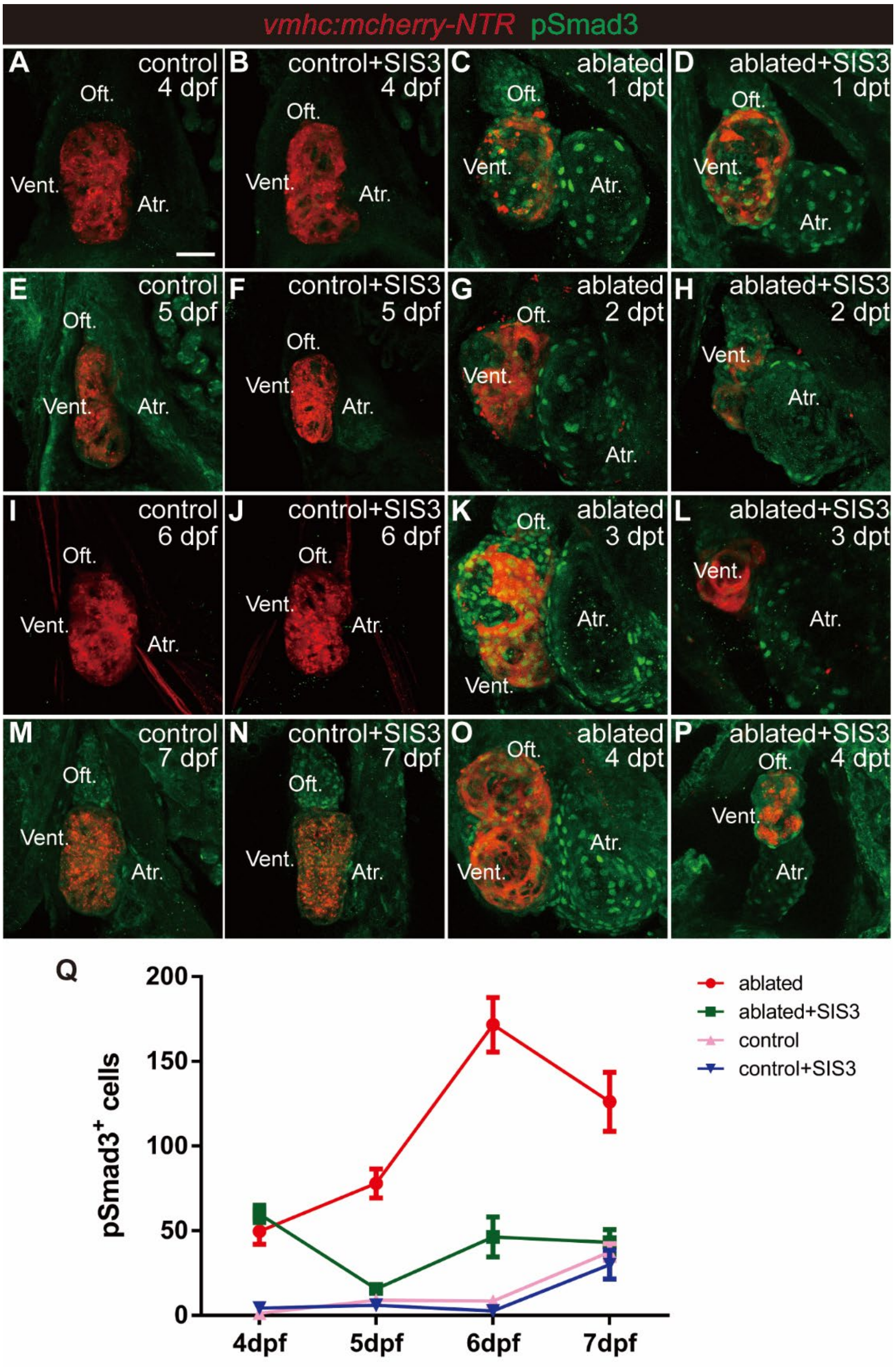

Supplementary figure 5. Dynamic changes of phospho-Smad3 signal during cardiac regeneration.

(A-P) Representative immunostaining images of *Tg(vmhc:mCherry-NTR)* hearts showed dynamic upregulation of phospho-Smad3 signal in the ablated hearts at various time points can be abolished by SIS3 treatment.

(Q) Quantification of the dynamic changes of phospho-Smad3 positive cell number in the control or ablated hearts without or with SIS3 treatment at 4 dpf (N=12, 14, 6, 5 for ablated, ablated+SIS3, control, control+SIS3, respectively), 5 dpf (N=11, 8, 8, 6), 6 dpf (N=6, 8, 8, 4) and 7dpf (N=11, 9, 8, 11).

Scale bar, 50 μm. dpf, days post fertilization; dpt, days post treatment; atr., atrium; oft., out flow tract; vent., ventricle.

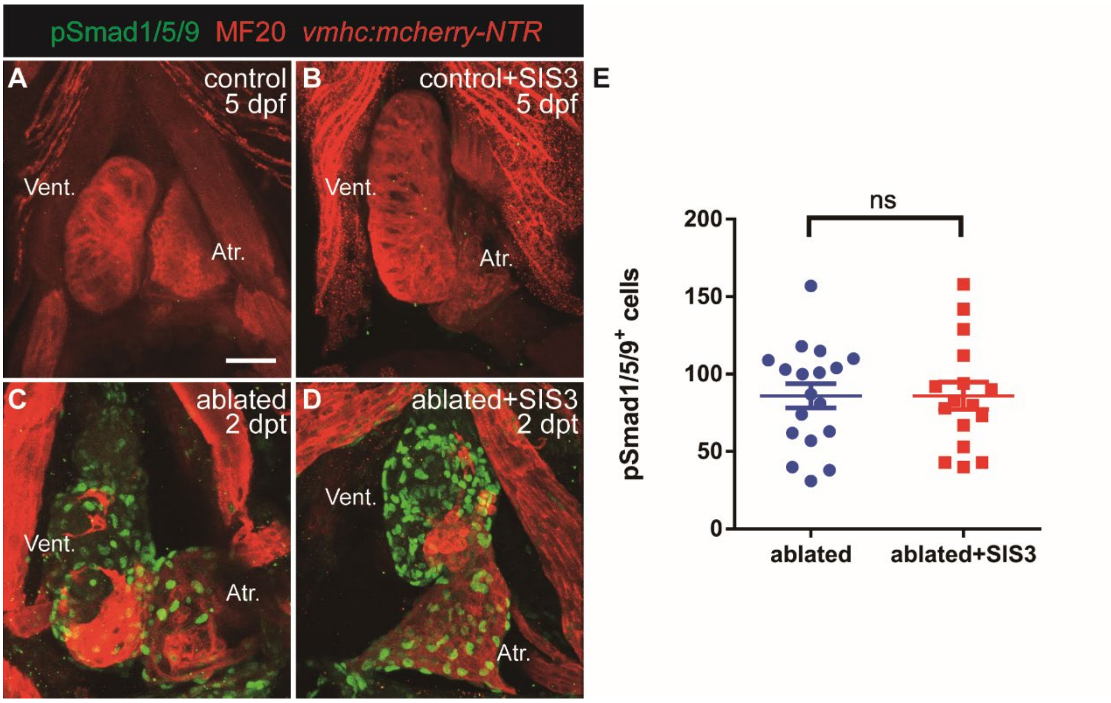

Supplementary figure 6. SIS3 treatment has no effect on Smad1/5/9 phosphorylation.

(A-D) Representative immunostaining images of *Tg(vmhc:mCherry-NTR)* hearts showed phospho-Smad1/5/9 signal was increased in the ablated hearts (C) but was not affected by SIS3 treatment (D) at 5 dpf/2 dpt. Green, anti-pSmad1/5/9; red, MF20 (anti-MHC).

(E) Quantification of phospho-Smad1/5/9 positive cell number in the ablated hearts without or with SIS3 treatment at 5 dpf/2 dpt. N=18 and 16, respectively. Mean ± s.e.m., Student's t-test, two-tailed, ns, non-significant.

Scale bar, 50 μm. dpf, days post fertilization; dpt, days post treatment; atr., atrium; vent., ventricle.

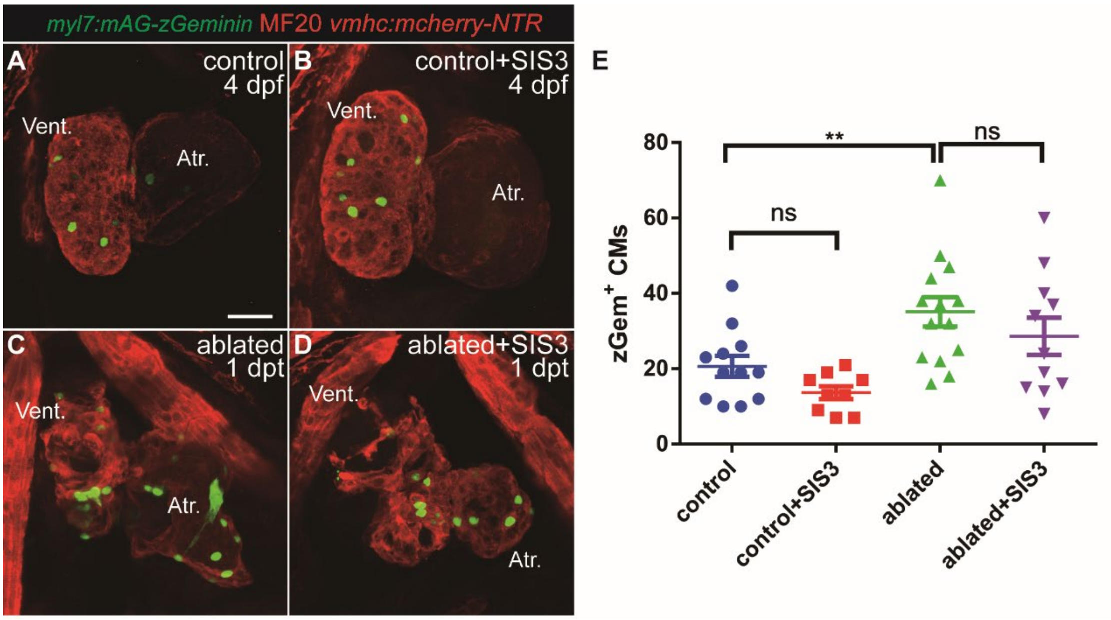

Supplementary figure 7. zGeminin positive CM number after 24-hour SIS3 treatment.

(A-D) Representative fluorescence images of *Tg(vmhc:mCherry-NTR; myl7:mAG-zGeminin)* larvae with immunostaining of MF20 (anti-MHC) at 4 dpf/1 dpt showed zGeminin positive CMs in the control or ablated hearts without or with 24-hour SIS3 treatment.

(E) Quantification of $zGem^+$ CM number in the control or ablated hearts without or with SIS3 treatment at 4 dpf/1 dpt. N=12, 9, 14, 11, respectively. Mean ± s.e.m., Student's t-test, two-tailed, ns, non-significant, **, $P<0.01$.

Scale bar, 50 μm. dpf, days post fertilization; dpt, days post treatment; atr., atrium; vent., ventricle.

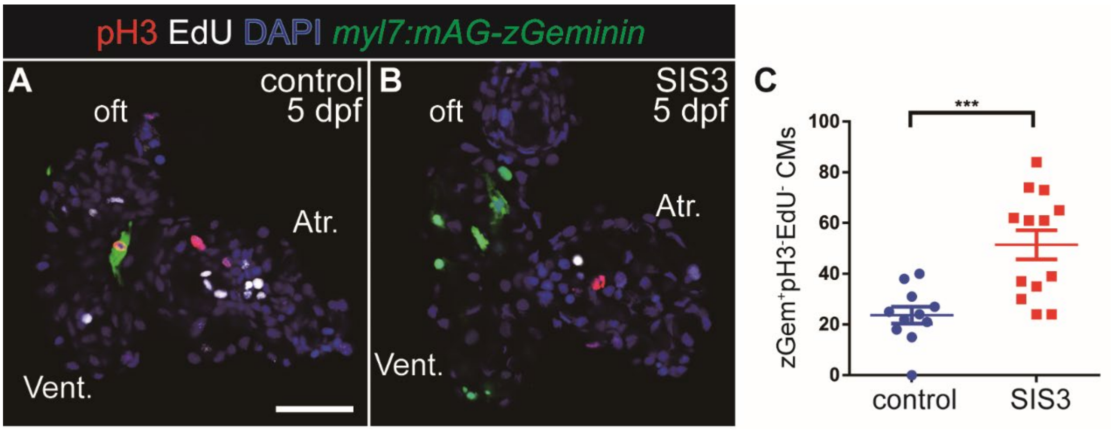

Supplementary figure 8. The number of zGem^{+}/pH3^{-}/EdU^{-} CMs is increased after 48-hour SIS3 treatment.

(A-B) Representative fluorescence images of *Tg(myl7:mAG-zGeminin)* hearts with immunostaining of phospho-histone H3 (red), EdU (white) and DAPI (blue) in the control hearts without or with 48-hour SIS3 treatment at 5 dpf.

(C) Quantification of zGem^{+}/pH3^{-}/EdU^{-} CM number in the control hearts without or with 48-hour SIS3 treatment at 5 dpf. The zGem^{+}/pH3^{-}/EdU^{-} CM number is defined as the total number of zGem^{+} CM subtracted by the numbers of zGem^{+}/pH3^{+} CM and zGem^{+}/EdU^{+} CM in the same heart. N=11 and 13, respectively. Mean ± s.e.m., Student's t-test, two-tailed, ***, P<0.001.

Scale bar, 20 µm. dpf, days post fertilization, dpt, days post treatment; atr., atrium; oft., out flow tract; vent., ventricle.

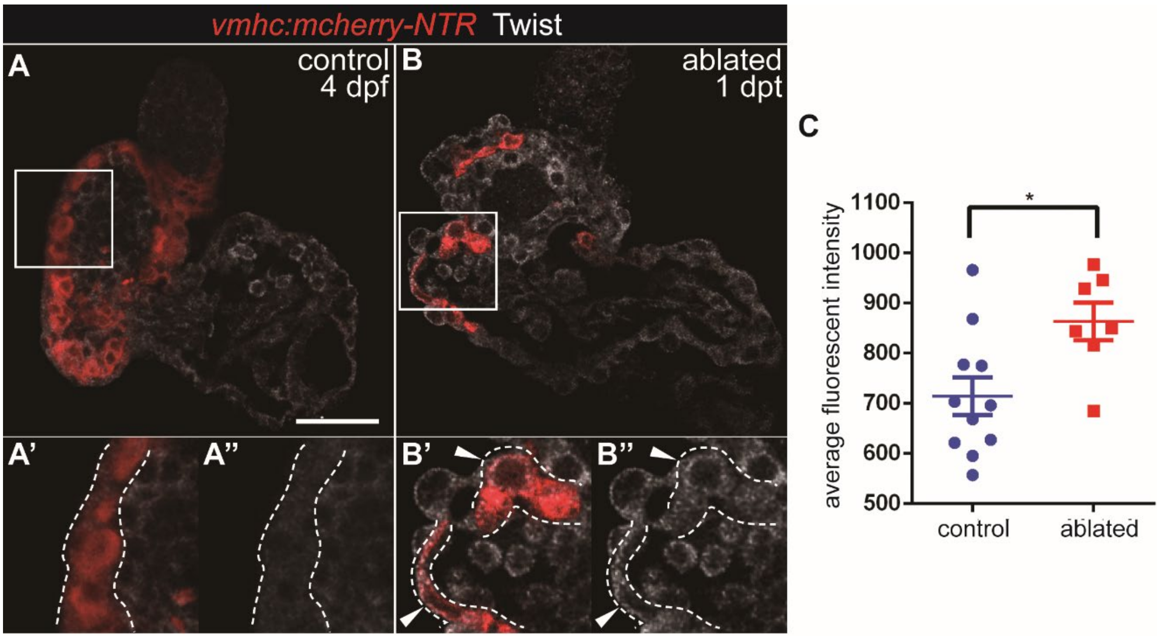

Supplementary figure 9. Twist is upregulated during cardiac regeneration.

(A, B) Representative immunostaining images of *Tg(vmhc:mCherry-NTR)* hearts showed Twist was upregulated in the ablated hearts compared to that in the control hearts at 4 dpf/1 dpt. A', B', enlargement of box area in A, B; A", B", white channel only. Dashed lines outline myocardial layer, arrowheads point to high Twist-expressed CMs.

(C) Quantification of average fluorescence intensity of Twist immunostaining in the control and ablated hearts at 4 dpf/1 dpt. N=11 and 7, respectively. Mean ± s.e.m., Student's t-test, two-tailed, *, $P<0.05$.

Scale bar, 20 µm. dpf, days post fertilization; dpt, days post treatment.

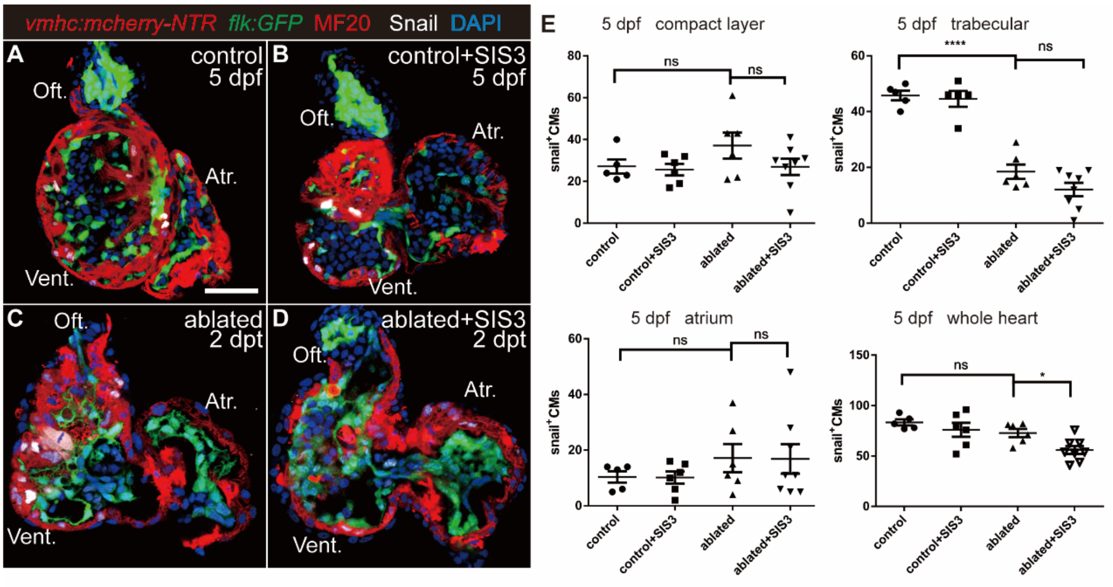

Supplementary figure 10. Smad3 inhibition reduces Snail positive CM number during regeneration.

(A-D) Representative fluorescence images of *Tg(vmhc:mCherry-NTR; flk:GFP)* hearts with immunostaining of Snail (white), MF20 (red) and DAPI (blue) in the control or ablated hearts with or without SIS3 treatment at 5 dpf/2 dpt.

(E) Quantification of Snail positive CM number in the compact layer, trabeculae, atrium and whole heart of control, control+SIS3, ablated and ablated+SIS3 larvae at 5 dpf/2 dpt. N=5, 6, 6, 8, respectively. Mean ± s.e.m., Student's t-test, two-tailed, ns, non-significant, *, $P<0.05$, ****, $P<0.0001$.

Scale bar, 50 µm. dpf, days post fertilization, dpt, days post treatment; atr., atrium; oft., out flow tract; vent., ventricle.
